# Seasonal and Technological Shifts of the WHO Priority Multi-Resistant Pathogens in Municipal Wastewater Treatment Plant and Its Receiving Surface Water: A Case Study

**DOI:** 10.3390/ijerph19010336

**Published:** 2021-12-29

**Authors:** Łukasz Jałowiecki, Jakub Hubeny, Monika Harnisz, Grażyna Płaza

**Affiliations:** 1 Environmental Microbiology Unit, Institute for Ecology of Industrial Areas, 40-844 Katowice, Poland; g.plaza@ietu.pl; 2 Department of Engineering of Water Protection and Environmental Microbiology, Faculty of Geoengineering, University of Warmia and Mazury, 10-719 Olsztyn, Poland; jakub.hubeny@uwm.edu.pl (J.H.); monikah@uwm.edu.pl (M.H.)

**Keywords:** wastewater, multi-antibiotic resistant pathogens, WHO priority pathogens list, metagenome analysis

## Abstract

The present study was focused on the identification of multi-resistant bacteria from the WHO priority pathogens list in the samples taken from different stages of the full-scale municipal wastewater treatment plant and receiving water. Additionally, the seasonal variations of the selected multi-resistant pathogens were analyzed in the samples. In order to the aim of the study, the metagenomic DNA from the collected samples was isolated and sequenced. The samples were collected in three campaigns (spring, summer, autumn). Metagenomic DNA was isolated by the commercial kits, according to the manufacturer’s instruction. Illumina sequencing system was employed, and the R program was used to metagenomic analysis. It was found that the wastewater samples and receiving water contained the multi-resistant bacteria from the WHO priority pathogens list. The seasonal and technological variations affected the distribution of the pathogens in the wastewater. No effect of the effluent on the pathogens in the receiving water was observed. The results indicated that antibiotic-resistant “priority pathogens” from the WHO list are there in the waste- and receiving water. Technological process and seasons effected their distribution in the environment. Metagenomic analysis can be used as sufficient tool in microbiological and human health risk assessment.

## 1. Introduction

Hundreds of antibiotics have been discovered or developed over 70 years, starting their huge application in medicine, veterinary, and agriculture [1,2]. Due to unmonitored and overuse of antibiotics, antimicrobial resistance (AMR) has been recognized by WHO as a major threat to global health [3,4]. The main reasons of AMR increase are the growth of various microbial infections, as well as the overuse and over-prescription of antimicrobials [5]. Human activities are mainly responsible for high levels and prevalence of antimicrobial resistance, which is now considered a modern phenomenon [6,7]. As calculated, around 10 million deaths are suspected by 2050 to occur annually due to AMR, and the cost of the situation is estimated ~US $100 trillion in total [8].

Among the anthropogenic sources, effluents from urban wastewater treatment plants (WWTPs) are suspected to be the main source of antibiotics resistance [9]. In recent years, wastewater plants were considered “hot spots” not only for antibiotic resistance but also for bacterial pathogens [10,11,12,13,14]. In addition, WWTPs are places where human activities and the environment are linked, and the horizontal transfer of resistance determinants among environmental microorganisms and clinically relevant pathogens is facilitated.

Many new techniques in treatment plants (biological, chemical, and physical) or their combinations have been now adopted to remove microbes from wastewater [15]. Despite the new approaches used, the pathogenic microbes exist in treated wastewater, and they are still considered a potential hazard to human health and the environment [16,17,18]. At present, biological indicators, such as the total coliforms, fecal coliforms, and *Escherichia coli*, are used to assess the quality of water and evaluate potential health risks, neglecting the problem of multi-antimicrobial resistance and multi-resistant pathogens [19].

WHO has developed a priority pathogens list (PPL) of antibiotic-resistant bacteria that pose the greatest threat to human health [20]. The PPL defines the priority of 12 pathogens, based on resistance to the most popular and widely applicable antibiotics for treating multi-drug resistant bacteria, such as carbapenems, third-generation cephalosporins, vancomycin, methicillin, penicillins, or fluoroquinolones. Twelve multi-resistant bacteria, posing the greatest threat to human health, are categorized to three priority tiers: critical, high, and medium, in terms of their resistance to the selected antimicrobials [21]. The pathogens are listed in Table 1 by group and antibiotic resistance characteristics.

In this context, the purpose of this study was to identify the multi-resistant bacteria from the WHO priority pathogens list in the samples taken from different wastewater treatment unit processes and receiver surface water. Additionally, the seasonal variations of the selected pathogens were analyzed in the samples. Our intention was to answer the question of whether, and how, the technological process and seasons affected the distribution of pathogens. This research may help us in understanding the dissemination of pathogenic bacteria, in terms of their significance as microbiological indicators in microbiological risk assessment (MRA). In the future, the results could be useful in developing appropriate treatment systems and their proper management.

## 2. Materials and Methods

### 2.1. Description of WWTP and Sample Collection

The samples were collected in three seasons: summer (June 2018), autumn (November 2018), and spring (March 2019) from the full-scale municipal wastewater treatment plant in the south part of Poland (50°5′35.881 N; 19°3′32.202 E). The detailed description of technological process of WWTP was presented by Rolbiecki et al. [22]. In Table 2, some technological parameters are presented.

**Figure 1:**
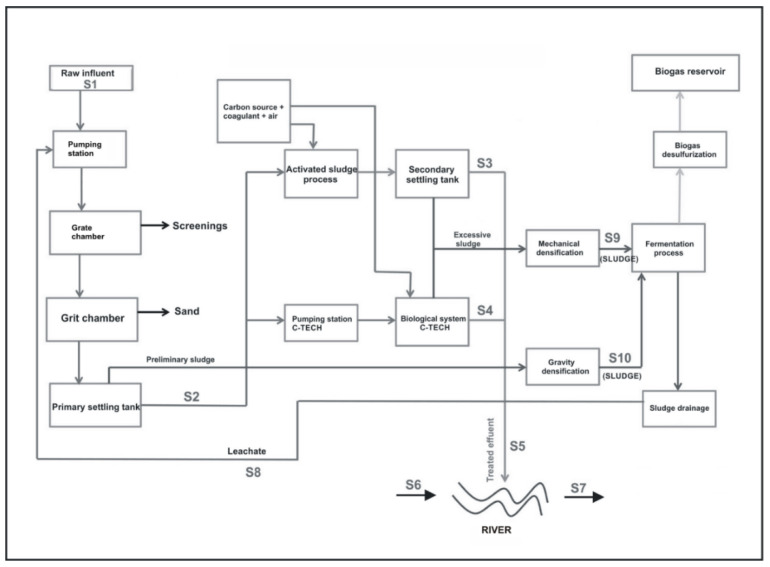
Scheme of wastewater treatment plant with sampling point.

During the sampling campaigns, 30 grab samples were collected in the sampling points presented in Figure 1. The sampling and transportation procedures used are described by Rolbiecki et al. [22] and Płaza et al. [23].

### 2.2. DNA Extraction and Illumina Sequencing

The wastewater samples were filtered in triplicate through a 0.22 μm micropore membrane (Whatman, Merck, Germany) and kept at −80 °C until DNA extraction. The Power Water kit (MoBio Laboratories Inc., Carlsbad, CA, USA) was used for isolation of metagenomic DNA (met DNA) from wastewater and river waters. Metagenomic DNA (met DNA) from sewage sludge samples was isolated by the Power Soil kit (MoBio Laboratories Inc., Carlsbad, CA, USA). All isolations were performed according to the manufacturer’s instruction. Quantity and quality of metDNA were determined by microspectrophotometry (BioSpectrometer, Eppendorf, Hamburg, Germany).

Extracted DNA samples were sent to Macrogen Inc. (Seoul, Korea) for library preparation and sequencing. An Illumina HiSeq sequencing system was used for sequencing.

### 2.3. Data Analysis

Sequencing results were uploaded to the MetaGenome Rapid Annotation Subsystems Technology (MG-RAST version 4.0.3) server as FASTQ files for analysis [24]. Details of the metagenomic analysis are presented in the paper by Płaza et al. [23]. The sequences were submitted to NCBI database, and they are under BioProject number ID: PRJNA666519.

Statistical analysis, in the form of PCA and Spearman rank correlation, were performed using Statistica v.13.3.

## 3. Results

In this paper, the technological and seasonal changes of antibiotic resistant priority pathogens from the WHO list were evaluated. Bacteria from the WHO priority pathogens list constituted from 2.15% of relative abundance of total identified bacteria in spring to 3.44% and 3.26% in autumn and summer, respectively. Twelve WHO priority pathogens were detected in all wastewater samples and the receiving surface water. The most abundant were pathogens belonging to priority 1, e.g., critical level in the waste- and receiving water. The dominant group of bacteria, from the WHO priority pathogens list, was *Enterobacteriaceae*. The average values of *Enterobacteriaceae* in all samples were 36.3%, 34.5%, and 23.7% in summer, autumn, and spring, respectively. *E. coli* and *Klebsiella pneumoniae* dominated in *Enterobacteriaceae*. The second most frequently isolated pathogen was *Acinetobacter baumannii*. The percentages of the bacteria were 18.6%, 17.5%, and 16.7% in summer, autumn, and spring, respectively. The third most frequently isolated pathogen was *Pseudomonas aeruginosa*. Among the rest of bacteria *Campylobacter* belonged to class of priority 2 (high), which was the most numerous. 

**Figure 2:**
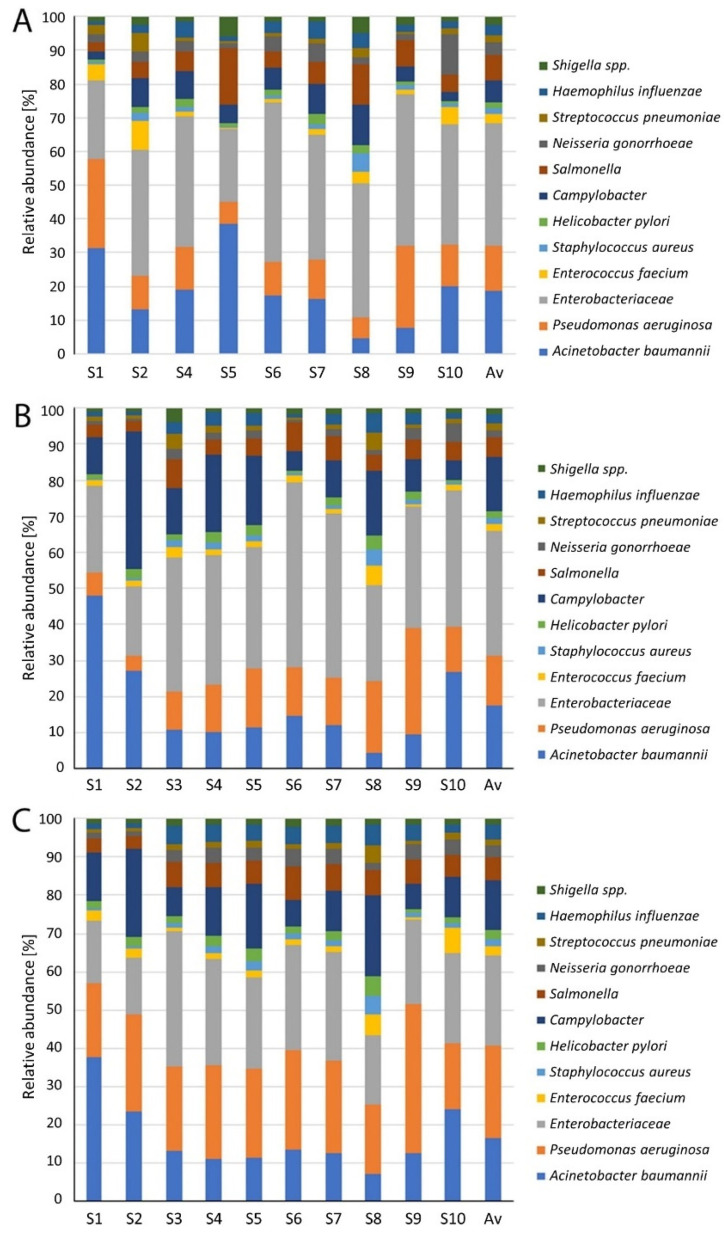
Technological and season changes of multi-antibiotic resistant pathogens from WHO list. (**A**) Summer (SUM); (**B**) autumn (AUT); (**C**) spring (SPR); Av—average.

**Figure 3:**
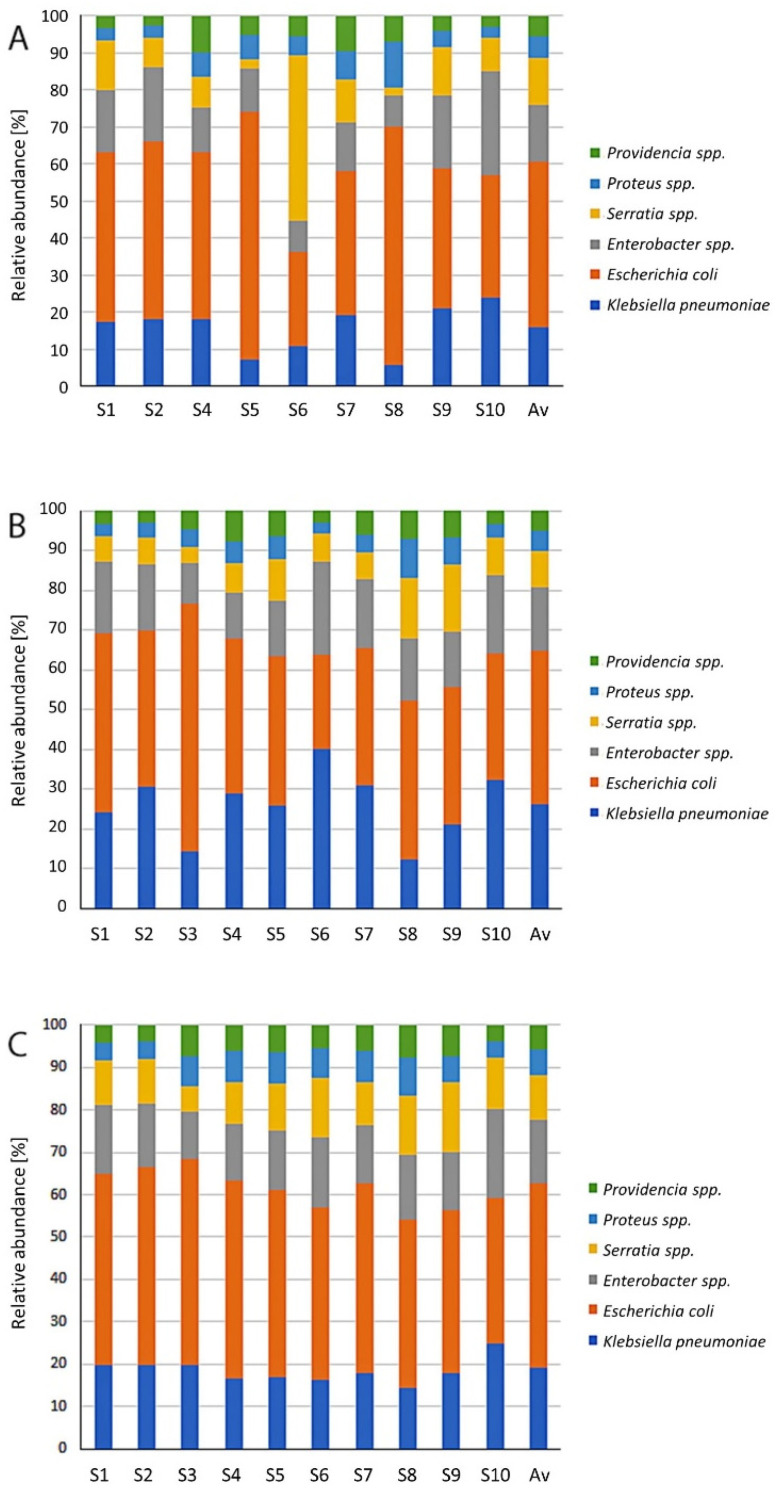
Technological and season changes of bacteria belonged to *Enterobacteriaceae*. (**A**) Summer (SUM); (**B**) autumn (AUT); (**C**) spring (SPR); Av—average.

**Figure 4:**
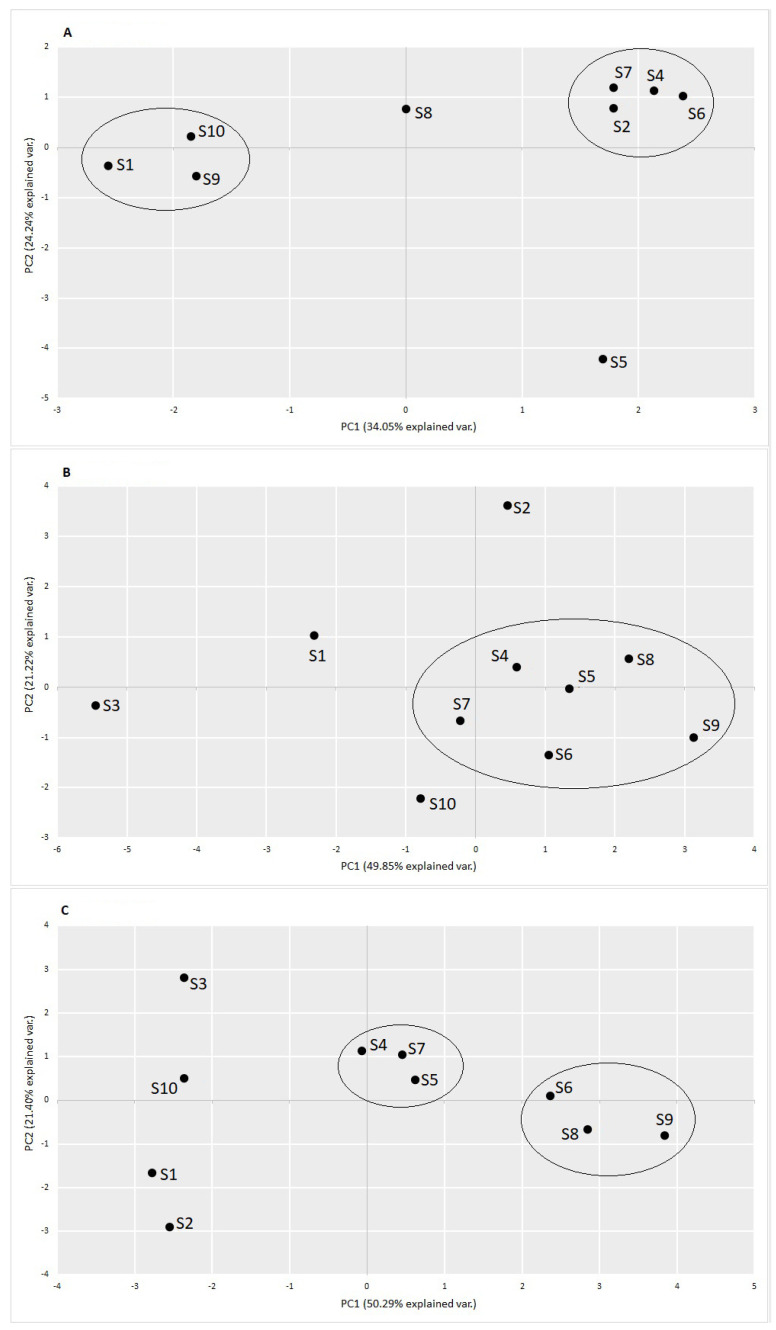
Principal component analysis (PCA) of the samples collected from different technological steps in various seasons on the variance-covariance matrix of the relative abundances of pathogens from the WHO multi-resistant pathogens list. The numbers in brackets describe the percentage of variance, explained by the first two components. (**A**) summer 2018; (**B**) autumn 2018; (**C**) spring 2019.

**Figure 5:**
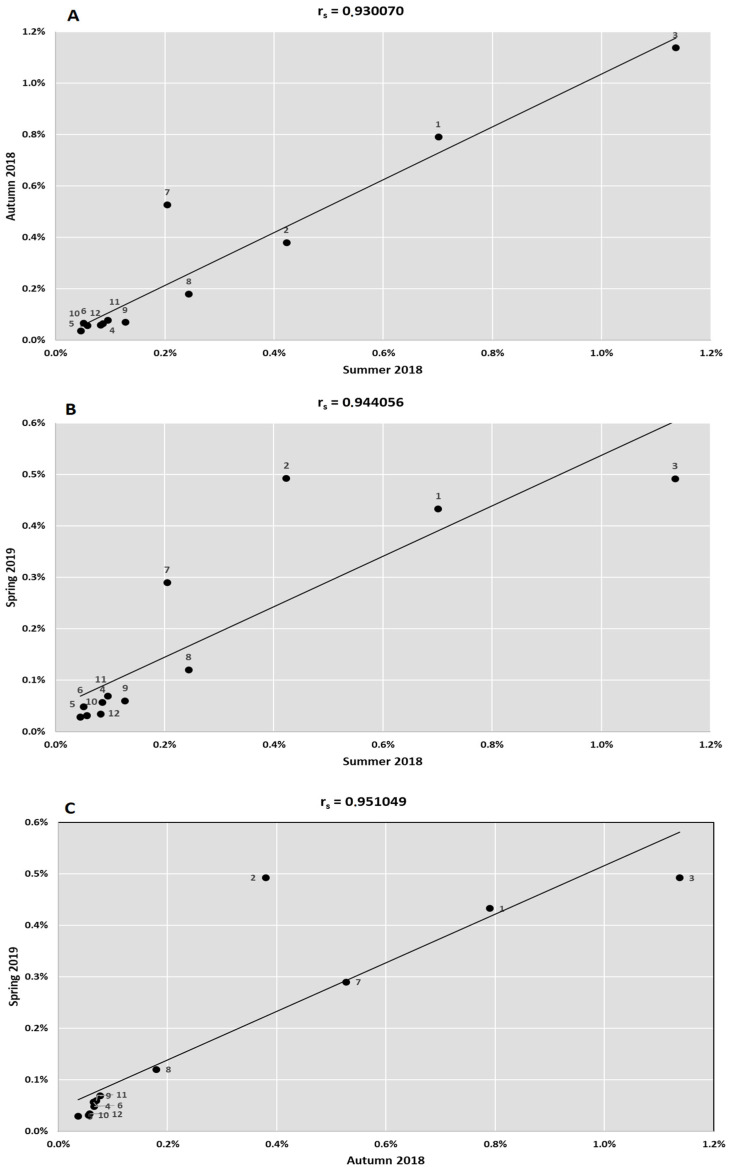
The Spearman’s rank-order correlation coefficient between the bacterial pathogens from the WHO list, occurring in various seasons (*p* < 0.01). 1—*Acinetobacter baumannii*; 2*—Pseudomonas aeruginosa*; 3—*Enterobacteriaceae*; 4*—Enterococcus faecium*; 5*—Staphylococcus aureus*; 6*—Helicobacter pylori*; 7*—Campylobacter* spp.; 8—*Salmonella* spp.; 9*—Neisseria gonorrhoeae*; 10*—Streptococcus pneumoniae*; 11*—Haemophilus influenzae*; 12*—Shigella* spp. (**A**) summer 2018; (**B**) autumn 2018; (**C**) spring 2019.

The revealed information on the changes of the pathogens in the different technological steps and seasons is summarized in Figure 2. In Figure 3, changes in the *Enterobacteriaceae* family are presented. The distribution of all dominant species varied greatly in different seasons and technological steps. Treated wastewater did not have a significant influence on the structure and distribution of pathogens in the downstream surface water. In the Figure 4 the results from the principle component analysis are presented. There was a difference between distribution of WHO priority pathogens in raw sewage (influent) and treated wastewater. The weak correlations were detected between the samples. All the tested samples contained twelve bacteria from the WHO priority pathogens list. In Figure 5, the correlations between the seasons are presented. In all analyses, the values of Spearman correlation index between seasons were high. It is suggested that environmental parameters, such as temperature differences, effect pathogen distribution in various seasons.

**Figure 6:**
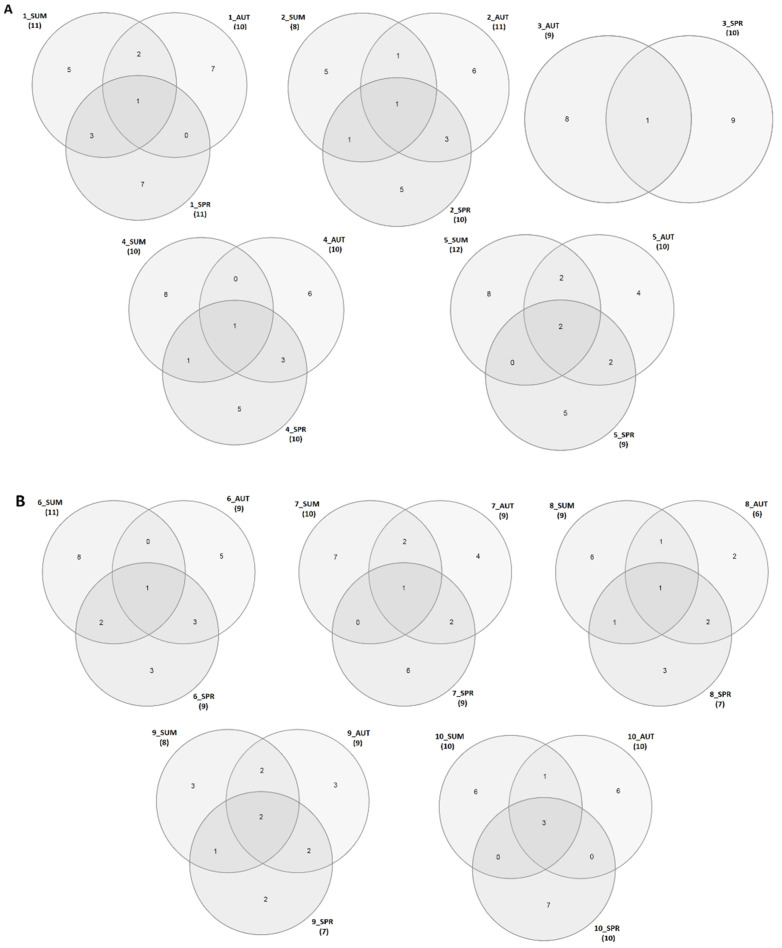
Venn diagrams illustrate the pathogens from WHO multi-resistant pathogens list in the samples collected from different technological steps. (**A**) Samples 1–5; (**B**) Samples 6–10; summer—SUM; autumn—AUT; spring—SPR.

In Figure 6A,B, the Venn diagrams for the selected technological steps and seasons are presented. *Venn diagram* worksheets present the relations between the abundance of pathogens in three seasons in the following technological steps. They graphically present how the distribution of pathogens in three seasons is different in the various technological steps.

## 4. Discussion

Based on the observed findings, the WHO priority multi-resistant pathogens were in wastewater and receiving surface waters. In the recent years, the number of pathogens with multi-drug resistance genes has significantly increased, and microbiological monitoring should be carried out in different environments, in order to prevent pollution and protect the environment and public health. Despite modern advances in wastewater technologies, treated effluents contain large amounts of various pollutants, including microbiological. The effluents are discharged into the environment, mainly into the receiving surface waters, consequently affecting public health directly or indirectly [25].

Generally, the microbial indicators are classified into three groups [26]: (i) general (process) microbial indicators, (ii) fecal indicators (such as *E. coli*), (iii) index organisms and model organisms. Presently, the following indicators of microbial contamination are used: total coliforms, enterococci, fecal streptococci, *Escherichia coli*, and *Clostridium perfringens*. Preferred indicators of fecal pollution are the enterococci [27]. These indicators are detected by the most of researches. Wen at al. [27] analyzed and compared water quality indicator systems in USA, Africa, and several countries in Asia and Europe. Currently, the bacterial indicators are the most popular in microbiological water quality monitoring, although several countries started to adopt the new methods for detecting other microbial pathogens, such as enteric viruses or protozoa [28].

Ajonina et al. [29] examined the microbiological quality of wastewater from the wastewater treatment plants in Hamburg City. As presented, the large amounts of coliform bacteria were found in treated wastewater and in River Elbe water. Marie and Lin [30] used the following bacterial indicators: *E. coli*, total coliforms, fecal coliforms, fecal streptococci, *Vibrio*, *Salmonella*, and *Shigella* for the evaluation of river water quality. Whereas Garrido-Perez et al. [31] evaluated the microbiological contamination of beach waters and sediments, using two indicators of fecal pollution: fecal coliforms and *Clostridium perfringens*. The occurrence of microbial indicators in waters is being reported by various researchers, but in most of the paper, there is no information about their antibiotic resistance. The characterization of the bacteria multi-antibiotic resistance has been neglected. Now, some epidemiological circumstances have changed this situation. Pérez-Rodríguez and Taban [32] reviewed the role of foods from animals (for example milk, meat, and poultry, etc.) as vehicles for multi-drug resistant pathogens and their role in the dissemination of antimicrobial resistances and novel characteristics, particularly multi-drug resistance.

However, most of the microbiological indicators are bacteria-specific, multi-antibiotic resistance; now, the multi-antibiotic resistance is a new characteristic of pathogens. Antimicrobial resistance (AMR) is huge public and health problem. As estimated, over 670,000 infections are caused annually by AMR pathogens in Europe. The cost of these infections is estimated to exceed 1 billion euros (ECDC, 2019). Most of the deaths are caused by pathogens, from which most of them are multi-drug resistant species, for example: *Escherichia coli*, *Acinetobacter* spp, *Pseudomonas aeruginosa*, *Klebsiella pneumoniae*, *Staphylococcus aureus*, and *Enterococcus* spp. Additionally, most of the infections are caused by these pathogens, and appropriate management, including antibiotic resistance of public health system, is essential.

In the review of Kakoullis et al. [33], the mechanisms of antibiotic resistance in pathogens, which are of great clinical important, were described. The author presented the resistance mechanisms of six pathogens, e.g., multi-drug resistant *Escherichia coli*, *Staphylococcus aureus*, *Pseudomonas aeruginosa*, *Enterococcus* spp., *Acinetobacter* spp., and *Klebsiella pneumoniae*. By the basic understanding the mechanisms of resistance, the clinicians can better comprehend and predict resistance patterns and subsequently select the most appropriate novel antimicrobial drugs for the pathogens or development of an effective vaccine [34,35].

In the study of Zaha et al. [36], the most commonly isolated pathogen was *Acinetobacter baumannii*, which were resistant to all the β–lactam antibiotics, including the carbapenems. Similar results were presented in the study of Handal et al. [37].

Fischbach and Walsh [38] have distinguished three classes of antibiotic-resistant pathogens, which are major threats to public health. First, methicillin-resistant *Staphylococcus aureus* (MRSA), which has high mortality rate. Pathogens from the second class, belonging to multidrug-resistant (MDR) and pan-drug-resistant (PDR) gram-negative bacteria. These strains of *Acinetobacter baumannii*, *Escherichia coli*, *Klebsiella pneumoniae*, and *Pseudomonas aeruginosa* are resistant to all antibiotic group: penicillins, cephalosporins, carbapenems, monobactams, quinolones, aminoglycosides, tetracyclines, and polymyxins. The third class belongs to strains of *Mycobacterium tuberculosis*.

Mhondoro et al. [39] noted that carbapenem-resistant *A. baumannii*, carbapenem-resistant *P. aeruginosa*, fluoroquinolone-resistant *Salmonella*, and ESBL producing *Enterobacteriaceae* were major health problems in Harare, Zimbabwe.

From the analysis conducted here, differential patterns in the distribution of WHO multi-resistant pathogens are clear, although the factors (technological steps and seasons) influencing the observed differences are yet to be fully explained. The effect of wastewater treatment processes on the fate of resistance is variable. As suggested by Karkman et al. [40], future research should examine whether WHO pathogens persist in downstream environments and urban water. Most of the WHO pathogens are important indicators for specific water purposes, such as agriculture and aquaculture wastewater and reuse.

Furthermore, the use of a metagenomic approach allowed us to identify pathogens in the collected samples. The sequence data can be also used to predict antibiotic resistance and virulence phenotypes. Detailed information on application of metagenomics approaches and other modern methods in pathogens detection is described in the literature [41,42,43,44]. So far, there are no reliable methods that could be used to detect pathogenic bacteria in the environmental samples, to the best of our knowledge. Despite the fact that numerous research has been done, new emerging pathogens or patterns and potential human risk from urban wastewater release are still uncertain. The lack of reporting standards of WHO pathogens also makes it is difficult to protect public health. Quantitative microbial risk assessment (QMRA) could be useful tool to evaluate the health risk assessment and describe scenarios for the spread of multi-resistant pathogens in the environment.

## 5. Conclusions

The study facilitated the evaluation of similarities and differences in composition of the pathogens from the WHO list, during the wastewater treatment process and seasons, and their distribution in effluent receiving water. Special attention was paid, in order to present the metagenomic analysis as a new tool in human health risk assessment. Twelve bacteria from the WHO priority pathogens list were detected in wastewater and receiving surface water. They disperse in the environment and disease transmission. WHO-listed bacteria pose a serious component of the threat of microbial contamination, in regard to public health and water quality, especially during water recycling and reuse processes. The process of the identification and detection of WHO-listed bacteria is an important step in the management of health and environmental risks associated with recycled water.

## Figures and Tables

**Table 1:** WHO priority pathogens list.

Categories	Bacteria	Antibiotic-Resistance
Priority 1: Critical	*Acinetobacter baumannii*	Carbapenem-resistant
	*Pseudomonas aeruginosa*	Carbapenem-resistant
	Enterobacteriaceae	Carbapenem-resistant, ESBL-producing
Priority 2: High	*Enterococcus faecium*	Vancomycin-resistant
	*Staphylococcus aureus*	Methicillin-resistant, vancomycin-resistant
	*Helicobacter pylori*	Clarithromycin-resistant
	*Campylobacter* spp.	Fluoroquinolone-resistant
	*Salmonella* spp.	Fluoroquinolone-resistant
	*Neisseria gonorrhoeae*	Cephalosporin-resistant, fluoroquinolone-resistant
Priority 3: Medium	*Streptococcus pneumoniae*	Penicillin-non-susceptible
	*Haemophilus influenzae*	Ampicillin-resistant
	*Shigella* spp.	Fluoroquinolone-resistant

**Table 2:** Some technological parameters of wastewater treatment plant and meteorological indicators.

Technological Parameters *	Unit	Wastewater	June 2018	Autumn 2018	March 2019
Flow	m^3^/month		960,077	722,516	793,234
Temperature	°C	Influent	19.5	10.5	14.5
		Effluent	21	19	13.5
pH		Effluent	7.2	7.2	7.2
COD	mg/L	Influent	963	672	970
		Effluent	36.5	30.5	35.0
BOD5	mg/L	Influent	435	290	340
		Effluent	4.8	4.7	6.0
Suspension	mg/L	Influent	525	310	455
		Effluent	5.8	6.3	7.2
N_TOT_	mg/L	Influent	106.1	78.1	84.2
		Effluent	10.4	7.9	6.2
N_NH4+_	mg/L	Influent	32.25	48.90	56.90
		Effluent	0.36	0.22	0.31
P_TOT_	mg/L	Influent	8.88	14.9	11.4
		Effluent	0.74	1.1	0.5
SRT	d		17	19	20
HRT	h		9	9	9
SS	kg/m^3^		4.5	5.0	5.5
Meterological parameters *					
Temperature	°C		20.4	4.5	6.1
Rainfall	mm		71	14	59

* Monthly average; abbreviations: HRT—hydraulic retention time; SS—suspended solids; SRT—solid retention time.

## Data Availability

The metagenome data, used in submitted paper #738158, are deposited in the NCBI Sequence Read Archive (SRA), under accessions NCBI SRA BioProject: PRJNA666519—https://www.ncbi.nlm.nih.gov/bioproject/PRJNA6665196 (accessed on 20 February 2021).

## References

[1] Dias D.A., Urban S., Roessner U. (2012). A historical overview of natural products in drug discovery. Metabolites.

[2] Rex J.H. (2014). ND4BB: Addressing the antimicrobial resistance crisis. Nat. Rev. Microbiol..

[3] WHO (2017). Global Priority List of Antibiotic-Resistant Bacteria to Guide Research, Discovery, and Development of New Antibiotics. https://www.who.int/medicines/publications/global-priority-list-antibiotic-resistant-bacteria/en/.

[4] WHO (2020). Antimicrobial Resistance. https://www.who.int/news-room/fact-sheets/detail/antimicrobial-resistance.

[5] Michael C.A., Dominey-Howes D., Labbate M. (2014). The antimicrobial resistance crisis: Causes, consequences, and management. Front. Public Health.

[6] Davies J., Davies D. (2010). Origins and evolution of antibiotic resistance. Microbiol. Mol. Biol. Rev..

[7] D’Costa V.M., King C.E., Kalan L., Morar M., Sung W.W.L., Schwarz C., Froese D., Zazula G., Calmels F., Debruyne R. (2011). Antibiotic resistance is ancient. Nature.

[8] Trotter A.J., Aydin A., Strinden M.J., O’Grady J. (2019). Recent and emerging technologies for the rapid diagnosis of infection and antimicrobial resistance. Curr. Opin. Microbiol..

[9] Rizzo L., Manaia C., Merlin C., Schwartz T., Dagot C., Ploy M.C., Michael I., Fatta-Kassinos D. (2013). Urban wastewater treatment plants as hotspots for antibiotic resistant bacteria and genes spread into the environment: A review. Sci. Total Environ..

[10] Cai L., Zhang T. (2013). Detecting human bacterial pathogens in wastewater treatment plants by a high-throughput shotgun sequencing technique. Environ. Sci. Technol..

[11] Kumaraswamy R., Amha Y.M., Anwar M.Z., Henschel A., Rodriguez J., Ahmad F. (2014). Molecular analysis for screening human bacterial pathogens in municipal wastewater treatment and reuse. Environ. Sci. Technol..

[12] Chu B.T.T., Petrovich M.L., Chaudhary A., Wright D., Murphy B., Wells G., Poretsky R. (2018). Metagenomics reveals the impact of wastewater treatment plants on the dispersal of microorganisms and genes in aquatic sediments. Appl. Environ. Microbiol..

[13] Huang K., Zhao F., Zhang X.-X., Ye L., Ren H., Zhang T., Mao Y., Ju F., Wang Y., Li B. (2018). Free-living bacteria and potential bacterial pathogens in sewage treatment plants. Appl. Microbiol. Biotechnol..

[14] Osunmakinde C.O., Selvarajan R., Mamba B.B., Msagati T.A.M. (2019). Profiling bacterial diversity and potential pathogens in wastewater treatment plants using high-throughput sequencing analysis. Microorganisms.

[15] Rajasulochana P. (2016). Comparison on efficiency of various techniques in treatment of waste and sewage water—A comprehensive review. Resour. Technol..

[16] Okoh A.I., Odjadjare E.E., Igbinosa E.O., Osode A.N. (2007). Wastewater treatment plants as a source of microbial pathogens in receiving watersheds. Afr. J. Biotechnol..

[17] Naidoo S., Olaniran A.O. (2013). Treated wastewater effluent as a source of microbial pollution of surface water resources. Int. J. Environ. Res. Public Heal..

[18] Lu X., Zhang X., Wang Z., Huang K., Wang Y., Liang W. (2015). Bacterial pathogens and community composition in advanced sewage treatment systems revealed by metagenomics analysis based on high-throughput sequencing. PLoS ONE.

[19] Lucena F., Duran A.E., Morón A., Calderón E., Campos C., Gantzer C., Skraber S., Jofre J. (2004). Reduction of bacterial indicators and bacteriophages infecting fecal bacteria in primary and secondary wastewater treatments. J. Appl. Microbiol..

[20] WHO (2015). Global Action Plan on Antimicrobial Resistance. http://www.who.int/antimicrobial-resistance/global-action-plan/en/.

[21] WHO (2017). Prioritization of Pathogens to Guide Discovery, Research and Development of New Antibiotics for Drug Resistant Bacterial Infections, Including Tuberculosis.

[22] Rolbiecki D., Harnisz M., Korzeniewska E., Jałowiecki Ł., Płaza G. (2020). Occurrence of fluoroquinolones and sulfonamides resistance genes in wastewater and sludge at different stages of wastewater treatment: A preliminary case study. Appl. Sci..

[23] Płaza G., Jałowiecki Ł., Głowacka D., Hubeny J., Harnisz M., Korzeniewska E. (2021). Insights into the microbial diversity and structure in a full-scale municipal wastewater treatment plant with particular regard to Archaea. PLoS ONE.

[24] Meyer F., Paarmann D., D’Souza M., Olson R., Glass E.M., Kubal M., Paczian T., Rodriguez A., Stevens R., Wilke A. (2008). The metagenomics RAST server—A public resource for the automatic phylogenetic and functional analysis of metagenomes. BMC Bioinform..

[25] De Lima I.R., Dos Santos L.U., Tosetto M.S., Franco R.M., Guimarães J.R. (2014). Urban water reuse: Microbial pathogens control by direct filtration and ultraviolet disinfection. J. Water Health.

[26] Ashbolt N.J., Grabow W.O.K., Snozzi M., Fewtrell L., Bartram J. (2001). Indicators of microbial water quality. Water Quality: Guidelines, Standards and Health.

[27] Wen X., Chen F., Lin Y., Zhu H., Yuan F., Kuang D., Jia Z., Yuan Z. (2020). Microbial indicators and their use for monitoring drinking water quality—A review. Sustainability.

[28] Jurzik L., Hamza I.A., Puchert W., Uberla K., Wilhelm M. (2010). Chemical and microbiological parameters as possible indicators for human enteric viruses in surface water. Int. J. Hyg. Environ. Health.

[29] Ajonina C., Buzie C., Rubiandini R.H., Otterpohl R. (2015). Microbial pathogens in wastewater treatment plants (WWTP) in Hamburg. J. Toxicol. Environ. Health Part A.

[30] Marie V., Lin J. (2018). Microbial indicators and environmental relationships in the Umhlangane River, Durban, South Africa. Open Life Sci..

[31] Garrido-Perez M.C., Anfuso E., Acevedo A., Perales-Vargas-Machuca J.A. (2008). Microbial indicators of faecal contamination in waters and sediments of beach bathing zones. Int. J. Hyg. Environ. Health.

[32] Pérez-Rodríguez F., Taban B.M. (2019). A State-of-Art Review on Multi-Drug Resistant Pathogens in Foods of Animal Origin: Risk Factors and Mitigation Strategies. Front. Microbiol..

[33] Kakoullis L., Papachristodoulou E., Chra P., Panos G. (2021). Mechanisms of antibiotic resistance in important gram-positive and gram-negative pathogens and novel antibiotic solutions. Antibiotics.

[34] ECDC (2020). Antimicrobial Resistance in the EU/EEA—AER for 2019.

[35] Banin E., Hughes D., Kuipers O.P. (2017). Bacterial pathogens, antibiotics and antibiotic resistance. FEMS Microbiol. Rev..

[36] Zaha D.C., Bungau S., Aleya S., Tit D.M., Vesa C.M., Popa A.R., Pantis C., Maghiar O.A., Bratu O.G., Furau C. (2019). What antibiotics for what pathogens? The sensitivity spectrum of isolated strains in an intensive care unit. Sci. Total Environ..

[37] Handal R., Qunibi L., Sahouri I., Juhari M., Dawodi R., Marzouqa H., Hindiyeh M. (2017). Characterization of carbapenem-resistant *Acinetobacter baumannii* strains isolated from hospitalized patients in Palestine. Int. J. Microbiol..

[38] Fischbach M.A., Walsh C.T. (2009). Antibiotics for Emerging Pathogens. Science.

[39] Mhondoro M., Ndlovu N., Donewell B., Juru T., Tafara G.N., Gerald S., Peter N., Mufuta T. (2019). Trends in antimicrobial resistance of bacterial pathogens in Harare, Zimbabwe, 2012–2017: A secondary dataset analysis. BMC Infect. Dis..

[40] Karkman A., Pärnänen K., Larsson D.G.J. (2019). Fecal pollution can explain antibiotic resistance gene abundances in anthropogenically impacted environments. Nat. Commun..

[41] Miller R.R., Montoya V., Gardy J.L., Patrick D.M., Tang P. (2013). Metagenomics for pathogen detection in public health. Genome Med..

[42] Ibekwe A.M., Leddy M., Murinda S.E. (2013). Potential human pathogenic bacteria in a mixed urban watershed as revealed by pyrosequencing. PLoS ONE.

[43] Ramírez-Castillo F., Loera-Muro A., Jacques M., Garneau P., Avelar-González F., Harel J., Guerrero-Barrera A. (2015). Waterborne pathogens: Detection methods and challenges. Pathogens.

[44] Ricchi M., Bertasio C., Boniotti M.B., Vicari N., Russo S., Tilola M., Bellotti M.A., Bertasi B. (2017). Comparison among the quantification of bacterial pathogens by qPCR, dPCR, and cultural methods. Front. Microbiol..

